# EMPOWER@HOME:CONNECTED - TESTING A PILOT GROUP INTERVENTION FOR DEPRESSION AND LONELINESS IN OLDER ADULTS

**DOI:** 10.1093/geroni/igad104.3299

**Published:** 2023-12-21

**Authors:** Jay Kayser, Skyla Turner, Xiaoling Xiang

**Affiliations:** University of Michigan, Ann Arbor, Michigan, United States; University of Michigan, Ann Arbor, Michigan, United States; University of Michigan, Ann Arbor, Michigan, United States

## Abstract

Homebound older adults experience high rates of depression, social isolation, and loneliness, which adversely impacts physical and mental well-being. Accessing psychotherapy to address these concerns can be logistically challenging and cost prohibitive. This study examined the feasibility and preliminary effect of a novel group-based internet cognitive-behavioral therapy program called Empower@Home:Connected. The intervention was implemented over nine consecutive weekly sessions in small virtual groups (4-6 participants). These sessions were facilitated by trained mental health professionals and incorporated psychoeducational material, cognitive-behavioral therapy exercises, and socialization. Measurements on primary and secondary outcomes were gathered before and after completion of the intervention. In this preliminary pilot trial (NCT05732740), participants averaged 68 years and of those that attended the first session (n = 25), nearly all completed the program (n = 24). Session attendance was high, with an average session attendance rate of 94.70%. Following program completion, participants had statistically significant reductions in depressive symptoms (Cohen’s d = 1.01). Participants also had reductions in loneliness that trended towards statistical significance (p = 0.07); changes in social isolation were not statistically significant. Of the participants, 21 out of 23 expressed satisfaction with the program and would recommend it to peers. For secondary outcomes, analyses revealed significant improvements in anxiety and health-related quality-of-life. In this pilot feasibility trial, Empower@Home:Connected reduced depressive symptoms among older adults. The high adherence and satisfaction rates suggest acceptability. While the intervention produced improvements on several measures, further exploration is warranted to determine long-term impact and efficacy relative to other existing interventions.

